# Glycan clock of ageing—analytical precision and time-dependent inter- and i-individual variability

**DOI:** 10.1007/s11357-024-01239-4

**Published:** 2024-06-14

**Authors:** Borna Rapčan, Manshu Song, Azra Frkatović-Hodžić, Tea Pribić, Jakov Vuk, Anđelo Beletić, Maja Hanić, Julija Jurić, Petra Tominac, Josip Milas, Vedrana Ivić, Sven Viland, Sara Bonet, Branko Šego, Marija Heffer, Wei Wang, Michael P. Snyder, Gordan Lauc

**Affiliations:** 1 Genos Ltd, Borongajska Cesta 83H, 10000 Zagreb, Croatia; 2 Centre for Precision Health, Edith Cowan University, Perth, WA 6027 Australia; 3 Faculty of Pharmacy and Biochemistry, University of Zagreb, Ante Kovačića 1, 10000 Zagreb, Croatia; 4 GlycanAge Ltd, Helix, 3 Science Square, The Catalyst, Newcastle Upon Tyne, NE4 5TG UK; 5 Department of Public Health, Faculty of Medicine Osijek, J. J, Strossmayer University of Osijek, Huttlerova 4, 31 000 Osijek, Croatia; 6 Department of Medical Biology and Genetics Osijek, J. J. Strossmayer Faculty of Medicine, University of Osijek, Huttlerova 4, 31 000 Osijek, Croatia; 7 Clinical Research Centre, The First Affiliated Hospital of Shantou University Medical College, Shantou, 515041 China; 8 School of Public Health, Shandong First Medical University & Shandong Academy of Medical Sciences, Jinan, 250117 Shandong China; 9 Beijing Key Laboratory of Clinical Epidemiology, School of Public Health, Capital Medical University, Beijing, 100069 China; 10 Department of Genetics, Stanford University, 450 Jane Stanford Way, Stanford, CA 94305 USA

**Keywords:** Ageing, Glycans, Biomarkers, IgG *N*-glycosylation, Time-dependent stability

## Abstract

Ageing is a complex biological process with variations among individuals, leading to the development of ageing clocks to estimate biological age. Glycans, particularly in immunoglobulin G (IgG), have emerged as potential biomarkers of ageing, with changes in glycosylation patterns correlating with chronological age.

For precision analysis, three different plasma pools were analysed over 26 days in tetraplicates, 312 samples in total. In short-term variability analysis, two cohorts were analysed: AstraZeneca MFO cohort of 26 healthy individuals (median age 20) and a cohort of 70 premenopausal Chinese women (median age 22.5) cohort monitored over 3 months. Long-term variability analysis involved two adult men aged 47 and 57, monitored for 5 and 10 years, respectively. Samples were collected every 3 months and 3 weeks, respectively. IgG *N*-glycan analysis followed a standardized approach by isolating IgG, its subsequent denaturation and deglycosylation followed by glycan cleanup and labelling. Capillary gel electrophoresis with laser-induced fluorescence (CGE-LIF) and ultra-performance liquid chromatography analyses were employed for glycan profiling. Statistical analysis involved normalization, batch correction, and linear mixed models to assess time effects on derived glycan traits.

The intermediate precision results consistently exhibited very low coefficient of variation values across all three test samples. This consistent pattern underscores the high level of precision inherent in the CGE method for analysing the glycan clock of ageing. The AstraZeneca MFO cohort did not show any statistically significant trends, whereas the menstrual cycle cohort exhibited statistically significant trends in digalactosylated (G2), agalactosylated (G0) and fucosylation (F). These trends were attributed to the effects of the menstrual cycle. Long-term stability analysis identified enduring age-related trends in both subjects, showing a positive time effect in G0 and bisected *N*-acetylglucosamine, as well as a negative time effect in G2 and sialylation, aligning with earlier findings. Time effects measured for monogalactosylation, and F remained substantially lower than ones observed for other traits.

The study found that IgG *N*-glycome analysis using CGE-LIF exhibited remarkably high intermediate precision. Moreover, the study highlights the short- and long-term stability of IgG glycome composition, coupled with a notable capacity to adapt and respond to physiological changes and environmental influences such as hormonal changes, disease, and interventions. The discoveries from this study propel personalized medicine forward by deepening our understanding of how IgG glycome relates to age-related health concerns. This study underscores the reliability of glycans as a biomarker for tracking age-related changes and individual health paths.

**Supplementary Information:**

The online version contains supplementary material available at 10.1007/s11357-024-01239-4.

## Introduction

Ageing is a multifaceted biological phenomenon characterised by intricate molecular, cellular, and organismal transformations. These changes manifest at disparate time-dependent scales among individuals. Notably, some individuals maintain robust health well into advanced chronological age, exhibiting a remarkably slow pace of ageing. On the contrary, others undergo accelerated ageing, leading to a reduction in life expectancy and an increased vulnerability to age-related illnesses. Consequently, several ageing clocks have been developed in recent years with the primary goal of estimating biological age. Glycans have been reported as biomolecules that undergo alterations associated with ageing as early as 1988 [1]. Studies have investigated glycans as potential biomarkers of biological ageing, including the serum *N*-glycan profile [2] and the *N*-glycan profile of immunoglobulin G (IgG) [3].

Glycans are small sugar moieties covalently attached to other complex biological molecules, produced under specific conditions of time, location, and environment [4]. Glycans can be found in all domains of life [5]. The activity and functions of glycoproteins, including the main molecule of humoral immunity, IgG, are significantly influenced by *N*-glycosylation [6]. Human IgG glycosylation pattern changes were found to be implicated in numerous physiological [7, 8] and pathophysiological states and processes [9–12]. IgG glycosylation is highly reproducible in certain physiological states [13].

Nevertheless, changes in glycosylation patterns have been repeatedly reported as a compelling and robust marker of biological ageing [3, 14, 15]. Much like with disease and lifestyle choices, *N*-glycosylation patterns alter with progressing chronological age [3, 16]. The majority of studies investigating IgG glycosylation alterations during the ageing process consistently reported that IgG glycosylation patterns exhibit distinct characteristics during early adulthood, with the highest abundance of digalactosylated (G2) structures and the lowest amount of agalactosylated (G0) structures [16, 17]. However, as individuals age, there is a discernible decline in galactosylation levels accompanied by an increase in G0, along with a rise in bisected *N*-acetylglucosamine (B). Monogalactosylation (G1) was shown to be stable during the ageing process [18, 19]. Alterations in core fucosylation (F) and sialylation (S) have also been observed during the ageing process. However, consensus regarding the direction of these changes remains elusive, although a more prevalent observation of a decrease in the abundance of S IgG structures during the ageing process [18]. IgG glycan reliability as a biomarker of ageing was demonstrated by explaining a substantial portion, up to 64%, of the variation in chronological age [3, 19].

The data about the short- and long-term stability of the glycan clock of ageing are substantial for its use as a biomarker for measuring biological age. Several studies examined the time-dependent stability of *N*-glycome in humans. Gornik et al. evaluated short-term variation through 5 days and found plasma *N*-glycome to be stable through that period in healthy individuals [15]. Hennig et al. showcased the remarkable stability of the plasma *N*-glycome over a span of several years. As a result of longitudinal sampling of the same person, it was possible to detect small changes in glycome composition mainly linked to lifestyle and environmental factors [20]. Day-to-day variability of IgG *N*-glycome has also been investigated among nine individuals and the glycome remained stable for 4 days [21].

Given the pivotal role of IgG *N*-glycosylation in both health and disease, this study seeks to address existing research gaps by evaluating the analytical precision of the Genos-GlycanAge IgG glycome profiling test and investigating the short- and long-term stability of individuals’ IgG *N*-glycome. To address this gap, the study has three objectives: first, to evaluate the intermediate precision of the test; second, to assess short-term inter-individual variability; and third, to explore long-term intra-individual variability. The exploration of the intermediate precision of the analytical method is performed through tetraplicate analysis of three distinct plasma pools over a 26-day period. Additionally, the study observes a group of young, healthy individuals continuously for 3 months and monitors another group of premenopausal women for a duration of 12 weeks to determine the short-term stability of IgG *N*-glycome. Finally, the study follows two individuals longitudinally: one for 5 years and the other for 10 years. These varying observation periods enable an evaluation of both short- and long-term stability of individuals’ IgG *N*-glycome profiles. The findings from this study could enhance our understanding of glycan dynamics and their potential utility as reliable indicators of ageing processes, filling a crucial knowledge void in the field of age-related research.

## Methods

### Participants and sample collection

To assess analytical precision, three different plasma pool samples collected at the Croatian Institute of Transfusion Medicine (CITM) were utilised with approval from the institute’s ethical committee. Each sample underwent analysis in tetraplicate across 26 plates, with each plate corresponding to a day of analysis, resulting in a total of 104 replicates per sample and 312 replicates overall.

In the initial phase of our short-term stability assessment, we analysed a specific subgroup of participants from the “AstraZeneca MFO” vaccination study conducted at the Faculty of Medicine, Osijek, Croatia. Within this young AstraZeneca MFO cohort (AMC), we selected 10 males, aged between 19 and 23 years, and 28 females, aged between 19 and 24 years with a median age of 20 for both groups, all designated as unvaccinated healthy controls. Individuals with confirmed pre-existing medical conditions and habits known to impact IgG glycosylation were excluded from the study based on medical examinations and a review of their medical records [7]. This screening process was implemented with the primary objective of establishing a baseline cohort of individuals devoid of any medical conditions that might introduce confounding variables into our subsequent analysis. Moreover, any participant who developed a condition that may affect glycosylation will be excluded from further participation in the study to maintain the integrity of our analyses. We gathered samples at four separate time intervals spread across a span of 90 days: initially at the beginning of the study and then on days 30, 60 and 90 thereafter.

The second set of samples (Chinese premenopausal women cohort, CPWC) in this study utilised analytical data on glycan analysis from a previous study [22]. These samples excluded individuals who were confirmed to be in menopause, had any prior illnesses, used oral contraceptives or hormonal medications, smoked cigarettes, consumed alcohol or were pregnant. These exclusions were mandated due to the known influence of these variables on the *N*-glycosylation patterns of IgG molecules [7, 23]. Unlike the previous approach, which incorporated a multiparameter analysis, this statistical examination focused solely on evaluating glycans as an individual marker. Furthermore, the emphasis was on studying glycans within the context of biological variation rather than specifically investigating their association with the menstrual cycle’s effects. The set of samples employed in this research study was sourced from a project entitled “The association between the glycosylation of IgG and the female menstrual cycle” performed in collaboration with the Capital Medical University in Beijing, China. The exclusion criteria encompassed individuals confirmed to be in a state of menopause, those with documented pre-existing medical conditions, and users of oral contraceptives or other hormonal substances or medications. Consequently, a total of 70 premenopausal women, ranging in age from 19 to 48 years, with a median age of 22.5, were considered eligible for inclusion in the study.

In the long-term variability experiment, two adult men aged 47 (for testing of 5-year stability) and 57 (for testing of 10-year stability) were enrolled as participants. In the 5-year experiment, samples were taken every 3 months, while during the 10-year, every 3 weeks, regardless of conditions that may affect the subject’s glycome. In the 5-year experiment, we refrained from collecting samples during heightened inflammation caused by COVID-19 due to its known effects on glycosylation [9]. This systematic approach allowed for the investigation of potential changes and patterns in the variables under consideration over an extended timeframe.

A blood sample was collected in tubes with ethylenediaminetetraacetic acid from each participant at every time point. Cohort subjects were sampled fasted in the mornings. The samples were centrifuged at room temperature at 1500 g to separate the plasma. The plasma was then stored at a constant − 20 ℃ to prevent unnecessary thawing cycles before further analysis.

### Sample preparation and CGE-LIF analysis

For the analytical precision analysis, AMC and long-term variability, the IgG isolation, IgG *N*-glycan release and labelling were performed using a Genos-Glycanage IgG glycome profiling kit (Genos, Osijek, Croatia) and subsequent capillary gel electrophoresis with laser-induced fluorescence (CGE-LIF) analysis was adapted from previously published protocols [24, 25]. The process of extracting IgG involved diluting 25 µL of subject plasma samples and three distinct plasma standards in tetraplicate, serving as technical replicates of a known, previously analysed, glycome. This dilution was carried out using a 1:7 ratio with a 1 × PBS buffer, which was prepared in-house. Additionally, blank samples containing ultrapure water, without any analyte, were included to monitor and control for potential cross-contamination. The diluted samples were resuspended and filtered through a wwPTFE filter plate with 0.45-µm pore size (Pall corporation, New York, NY, USA) using a vacuum manifold and pump (Pall corporation, New York, NY, USA). The filtered samples were transferred to a CIM® r-Protein G LLD 0.05 mL monolithic 96-well plate (Sartorius BIA Separations, Ajdovščina, Slovenia), where they underwent binding and subsequent washing steps with phosphate-buffered saline (1 × PBS) buffer (0.25 M NaCl, increased ionic strength, prepared in-house). Elution of the bound IgG was achieved by employing 0.1 M formic acid neutralised with ammonium bicarbonate buffer (Sigma-Aldrich, St. Louis, MO, USA). The eluted IgG fraction (20 µL) was dried and prepared for the subsequent steps in the protocol.

The dried IgG samples were consecutively treated with 1.66 × PBS, 0.5% sodium dodecyl sulphate (SDS) and 2% Igepal (Sigma-Aldrich, St. Louis, MO, USA/Invitrogen Thermo Fisher Scientific, Carlsbad, CA, USA), to denature the IgG, followed by incubation with 1.2U of the enzyme PNGase F (Promega, Madison, WI, USA) at 37 °C for 3 h to release its *N*-glycans. The released glycans were then labelled by mixing APTS (8-aminopyrene-1,3,6-trisulfonic acid) (Synchem, Felsberg, Germany) fluorescent dye with the reducing agent 2-picoline borane (Sigma-Aldrich, St. Louis, MO, USA) and subjected to a 16-h incubation at 37 °C.

After incubation, the labelling reaction was halted by the addition of 80% acetonitrile (ACN, Carlo Erba, Milan, Italy). The clean-up of the released fluorescently labelled IgG *N*-glycans was conducted using solid-phase extraction utilising Bio-Gel P-10 as a hydrophilic stationary phase. The entire sample volume was transferred to the filter plate containing the Bio-Gel P-10. The excess label and reducing agent were removed by five washes with 80% ACN/100 mM triethylamine (Sigma-Aldrich, St. Louis, MO, USA), followed by three washes with 80% ACN. Finally, APTS labelled IgG *N*-glycans were eluted in ultra-pure water.

For CGE-LIF analysis, 3 µL of purified IgG *N*-glycans combined with 7 µL of Hi-Di Formamide were analysed using an ABI3500 Genetic Analyzer (Thermo Fisher Scientific, Waltham, MA, USA) equipped with a 50-cm long 8-capillary array filled with POP-7 polymer as a separation matrix. Run parameters were set as follows: run time 1000 s, injection time 12 s, injection voltage 15 kV, run voltage 15 kV, and oven temperature 60 °C. The resulting electropherograms were manually integrated into 27 glycan peaks using the Empower 3 software (Waters, Milford, MA, USA) [28]. The amount of glycan structures in a peak was expressed as a percentage of the total integrated area (total area normalisation). In addition, six derived glycan traits were calculated for glycans with shared structural features [13, 26].

### Sample preparation and UPLC analysis

The CPWC followed the adapted protocol reported by Pučić et al. and Akmačić et al. [24, 26]. As described in CGE-LIF preparation, IgG was extracted from plasma samples using the same protein G plate. The analysis was controlled by utilizing four replicates of a single previously analysed plasma standard and two blanks, as previously described. The isolated IgG was then subjected to denaturation by introducing SDS and incubating at 65 ℃. To counteract the excess SDS, Igepal-CA630 was employed. The release of IgG *N*-glycans was achieved by adding PNGase F in 1 × PBS and subsequently incubating overnight at 37 ℃. The released *N*-glycans were tagged with a fluorescent 2-aminobenzamide (2-AB) dye (Merck, Darmstadt, Germany). The unbound label and reducing agent were removed from the samples using hydrophilic interaction liquid chromatography solid-phase extraction (HILIC-SPE). Finally, the IgG *N*-glycans were eluted with ultrapure water and stored at –20 ℃ until needed.

For the separation of labelled *N*-glycans, HILIC (hydrophilic interaction liquid chromatography) was performed using a Waters Acquity H class ultra-performance liquid chromatography (UPLC) system (Waters, Milford, MA, USA). An Acquity UPLC Glycan BEH chromatography column (Waters, Milford, MA, USA) was utilised to separate the *N*-glycans. Solvent A consisted of 100 mmol/L ammonium formate at pH 4.4, while solvent B was 100% ACN. Samples were maintained at 10 ℃ before injection into the column, and the separation itself took place at 60 ℃. The separation method involved a linear gradient ranging from 75 to 62% ACN at a flow rate of 0.40 mL/min during a 27-min analytical run. The system was controlled using the Empower 3 software.

The chromatograms were divided into 24 Glycan peaks (GP1–GP24) using the same Empower 3 software [26]. The abundance of glycans within each peak was expressed as a percentage of the total integrated area (% area). In addition, six derived glycan traits were calculated for glycans with shared structural features [26].

### Statistical analysis

A custom Python script was used for the calculation of intermediate precision [27]. In the cohorts of AMC and CPWC, the glycan data were initially normalised and batch-corrected to remove experimental variation and facilitate sample comparison. Total area normalisation was performed on the area under the peaks, followed by log transformation and batch correction using the ComBat method as implemented in the “sva” package [28] in R [29]. Subsequently, the glycan peak values were transformed back to the original scale prior to the calculation of the derived glycan traits. Glycan trait values were transformed by inverse rank transformation to normality to obtain values with a mean of 0 and a standard deviation of 1. The approach for both the 5-year and 10-year samples followed the established pattern. First, normalisation was performed, where total area normalisation was applied to the area under the peaks, followed by log transformation. Glycan traits were then calculated as previously described [25, 26] (Additional file 1: Table S1).

The time effect on the derived glycan traits was tested separately in males and females using the linear mixed model, where time was modelled as a fixed effect. Figures and time effects for all analyses were generated and calculated in Python [27] along with Matplotlib [30], Seaborn [31] and Pandas [32] packages.

## Results

### Intermediate precision

Between-run or intermediate precision, which assesses the reproducibility of results over multiple runs, was evaluated using three different samples analysed across 26 plates in tetraplicate for the CGE-LIF method. Tables 1, 2, and 3 contain CVs (coefficients of variation) calculated for each glycan peak (1 through 27) and for derived glycan traits G0, G1, G2, S, B and F. Notably, the CVs for glycan peaks generally fell under 10%. GP20 showed the greatest variation among all glycan peaks, while GP14 and GP1 were notable exceptions, consistently showing CVs over 10%. When examining derived glycan trait variation, all traits showed CVs lower than 3%, with the S trait consistently showing the highest variation (2.24–2.75%), while the trait *F* showed the lowest variability, with CVs ranging from 0.21 to 0.28% (Tables 1, 2, and 3).

**Table 1:** The table presents the results of between-run precision analysis for standard 4, comprising various electropherogram peaks (i.e., directly measured glycan traits)

Glycan trait	x̄̄	*S*_*b*_	CV
GP1	0.55178	0.06442	11.67558
GP2	0.23598	0.01753	7.42890
GP3	2.49692	0.13954	5.58867
GP4	1.72242	0.10966	6.36666
GP5	0.15861	0.00947	5.97079
GP6	0.05235	0.00394	7.53482
GP7	0.26006	0.00684	2.62925
GP8	2.19150	0.04903	2.23728
GP9	0.29275	0.01205	4.11756
GP10	0.87814	0.05814	6.62112
GP11	0.26257	0.01283	4.88715
GP12	10.59108	0.17319	1.63528
GP13	2.32325	0.04467	1.92273
GP14	0.45787	0.08707	19.01597
GP15	17.75626	0.29415	1.65658
GP16	0.51105	0.03594	7.03281
GP17	0.31855	0.01611	5.05640
GP18	2.38132	0.00000	0.00000
GP19	0.26259	0.01089	4.14550
GP20	0.39808	0.10490	26.35179
GP21	19.82180	0.12608	0.63607
GP22	11.07802	0.11211	1.01196
GP23	4.62238	0.07290	1.57712
GP24	0.56735	0.00048	0.08507
GP25	0.14153	0.00974	6.88252
GP26	18.27007	0.35280	1.93105
GP27	1.39572	0.06021	4.31382
G0	20.59545	0.32694	1.58746
G1	37.97791	0.21225	0.55888
G2	19.80732	0.41745	2.10756
S	22.01741	0.59528	2.70366
B	14.77107	0.11053	0.74828
F	95.47815	0.20490	0.21460

Each glycan peak (GP) or derived glycan trait (i.e., glycosylation trait) is identified in the first column, followed by the grand mean (x̄̄), representing the average measurement across all runs, the standard deviation between groups (*S*_*b*_), indicating the variability between different experimental runs or batches, and the coefficient of variation (CV), expressing the between-run precision as a percentage. The corresponding letters (G0, G1, G2, *S*, *B*, *F*) represent distinct glycosylation traits: G0 for agalactosylation, G1 for monogalactosylation, G2 for digalactosylation, *S* for sialylation, *B* for bisecting *N*-acetylglucosamine, and *F* for core fucosylation in protein structures

**Table 2:** The table presents the results of between-run precision analysis for standard 5, comprising various electropherogram peaks (i.e., directly measured glycan traits)

Glycan trait	x̄̄	*S*_*b*_	CV
GP1	0.74127	0.08522	11.49711
GP2	0.30312	0.02647	8.73124
GP3	1.51147	0.09810	6.49043
GP4	1.65465	0.11243	6.79498
GP5	0.20889	0.01339	6.41001
GP6	0.04635	0.00385	8.30991
GP7	0.24067	0.00784	3.25609
GP8	1.76788	0.05528	3.12696
GP9	0.29028	0.00846	2.91285
GP10	0.94231	0.06915	7.33781
GP11	0.29301	0.00968	3.30328
GP12	6.72031	0.12628	1.87912
GP13	2.55097	0.04109	1.61082
GP14	0.64710	0.09990	15.43849
GP15	23.97806	0.33387	1.39240
GP16	0.44443	0.02536	5.70591
GP17	0.41783	0.02389	5.71667
GP18	3.98728	0.04582	1.14915
GP19	0.37449	0.01000	2.66971
GP20	0.47931	0.09729	20.29835
GP21	20.23916	0.14977	0.74002
GP22	11.09889	0.09277	0.83581
GP23	5.19449	0.05133	0.98808
GP24	0.70239	0.00882	1.25562
GP25	0.17065	0.01150	6.74164
GP26	13.54469	0.26104	1.92722
GP27	1.45005	0.08585	5.92065
G0	28.61244	0.38844	1.35758
G1	39.43031	0.24477	0.62076
G2	15.16539	0.34002	2.24208
S	17.27117	0.52706	3.05166
B	17.80750	0.27406	1.53904
F	94.64096	0.26649	0.28158

Each glycan peak (GP) or derived glycan trait (i.e., glycosylation trait) is identified in the first column, followed by the grand mean (x̄̄), representing the average measurement across all runs; the standard deviation between groups (*S*_*b*_), indicating the variability between different experimental runs or batches; and the coefficient of variation (CV), expressing the between-run precision as a percentage. The corresponding letters (G0, G1, G2, S, B, F) represent distinct glycosylation traits: G0 for agalactosylation, G1 for monogalactosylation, G2 for digalactosylation, S for sialylation, B for bisecting *N*-acetylglucosamine and F for core fucosylation in protein structures

**Table 3:** The table presents the results of between-run precision analysis for standard 6, comprising various electropherogram peaks (i.e., directly measured glycan traits)

Glycan trait	x̄̄	*S*_*b*_	CV
GP1	0.48855	0.04958	10.14813
GP2	0.27190	0.02621	9.63988
GP3	1.98800	0.12907	6.49246
GP4	1.97909	0.13060	6.59917
GP5	0.16456	0.00982	5.97012
GP6	0.03578	0.00526	14.70258
GP7	0.18830	0.00688	3.65463
GP8	1.55040	0.04856	3.13190
GP9	0.31241	0.01120	3.58647
GP10	0.87949	0.04480	5.09431
GP11	0.33641	0.01450	4.31158
GP12	7.98962	0.12962	1.62233
GP13	3.62573	0.09127	2.51728
GP14	0.59084	0.10994	18.60684
GP15	21.22035	0.28272	1.33231
GP16	0.47958	0.03157	6.58276
GP17	0.35701	0.02405	6.73661
GP18	3.64434	0.03545	0.97280
GP19	0.35736	0.01024	2.86464
GP20	0.39616	0.10016	25.28209
GP21	17.56454	0.12850	0.73156
GP22	11.33187	0.06913	0.61009
GP23	5.28527	0.06120	1.15787
GP24	0.76960	0.01297	1.68553
GP25	0.21029	0.01482	7.04591
GP26	15.89903	0.28134	1.76955
GP27	2.08351	0.13019	6.24873
G0	25.45553	0.31688	1.24485
G1	36.93758	0.23800	0.64434
G2	18.19282	0.40259	2.21291
S	19.81023	0.56217	2.83779
B	19.55050	0.33077	1.69189
F	95.11966	0.26471	0.27830

Each glycan peak (GP) or derived glycan trait (i.e., glycosylation trait) is identified in the first column, followed by the grand mean (x̄̄), representing the average measurement across all runs; the standard deviation between groups (*S*_*b*_), indicating the variability between different experimental runs or batches; and the coefficient of variation (CV), expressing the between-run precision as a percentage. The corresponding letters (G0, G1, G2, S, B, F) represent distinct glycosylation traits: G0 for agalactosylation, G1 for monogalactosylation, G2 for digalactosylation, S for sialylation, B for bisecting *N*-acetylglucosamine, and F for core fucosylation in protein structures

### Assessment of short-term stability

#### AMC

Given the known influence of the menstrual cycle on IgG glycosylation, we conducted separate statistical analyses for male and female participants to avoid any sex-specific effects that might affect short-term inter-individual variation evaluation in the cohort of healthy young individuals.

**Fig. 1:**
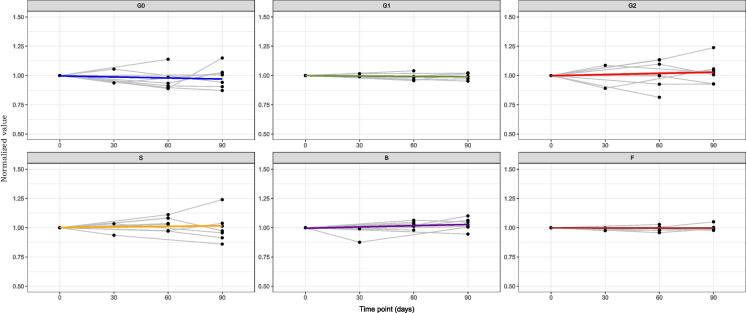
The plots illustrate the time-dependent trends for each derived glycan trait (G0, G1, G2, *S*, *B*, *F*) across the 10 men over the 90-day period. The *x*-axis represents time in days, while the *y*-axis displays the normalised values of relative abundance for each glycan trait. Data normalisation was conducted by dividing each data point value by its corresponding value at the initial point

For the 10 male young individuals, no significant changes were observed in any of the derived glycan traits during the 90-day period (Additional file 1: Table S2). Across the cohort, all glycan traits displayed a notable level of stability, with minimal variations on a group basis (Fig. 1). While a few individuals showed time-dependent changes at an individual level, the overall pattern indicated stability among male participants.

**Fig. 2:**
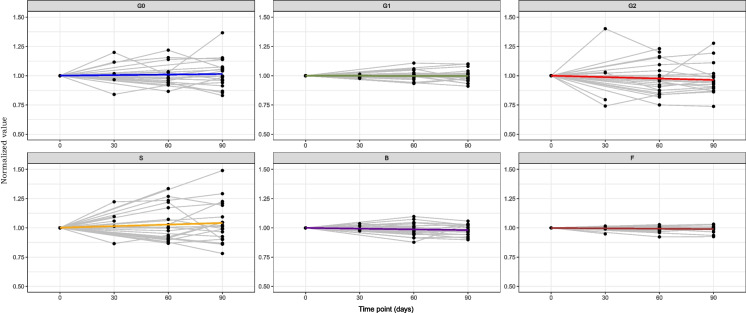
The plots illustrate the time-dependent trends for each derived glycan trait (G0, G1, G2, *S*, *B*, *F*) across the 28 women over the 90-day period. The *x*-axis represents time in days, while the *y*-axis displays the normalised values of relative abundance for each glycan trait. Data normalisation was conducted by dividing each data point value by its corresponding value at the initial point

In contrast, the cohort of 28 female participants exhibited more noticeable fluctuations in IgG glycosylation. Except for *F*, all glycan traits showed no statistically significant trends in their glycosylation patterns (Additional file 1: Table S3 and Fig. 2). Furthermore, after applying corrections for multiple testing, the statistical significance originally observed for variable *F* was dissipated.

#### CPWC

**Fig. 3:**
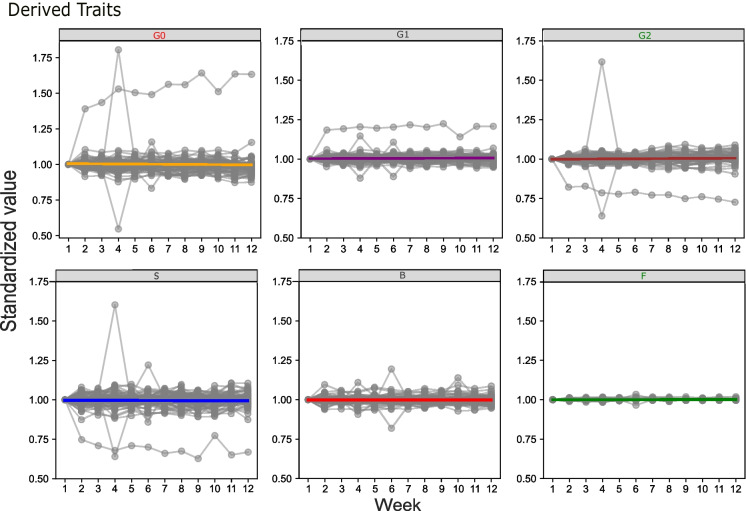
The plots illustrate the time-dependent trends for each derived glycan trait (G0, G1, G2, *S*, *B*, *F*) across the 70 women over the 12-week period. The *x*-axis represents time in weeks, while the *y*-axis displays the normalised values of relative abundance for each glycan trait. Data normalisation was conducted by dividing each data point value by its corresponding value at the initial point. Titles in green show statistically significant increase in glycan trait while the ones in red colour show statistically significant decrease in a glycan trait

To further examine the effect of short-term inter-individual variability of glycome, we employed another cohort of female participants experiencing the menstrual cycle, involving 70 individuals meticulously observed over 12 weeks. As we scrutinised the data, we observed analogous dynamics to those observed in AMC (Fig. 3).

Specifically, when examining the derived glycan traits of G1, B and S, we found that they remained notably stable over the entire duration of the study, with no statistically significant trends observed (Additional file 1: Table S4 and Fig. 3). On the other hand, G0, G2 and F showed statistically significant trends. G2 and F showed a positive time effect, while G0 exhibited a negative time effect. Notably, the time effects for F and G2 remained statistically significant even after applying corrections for multiple tests (Additional file 1: Table S4 and Fig. 3).

In the analysis of individual changes in glycan traits, we focused on the top 10% and bottom 10% of normalised results, representing values exceeding or falling below a specified threshold. This threshold, set at 9% above the initial measurement, enabled us to assess the frequency of extremes in our results. Our objective was to identify potential outliers that could signify measurement errors.

Notably, in one subject, we observed a substantial decrease after the first measurement of 25.3% in S and 18.4% in G2, accompanied by increases of 18.5% in G1 and 39% in G0. This trend persisted in subsequent measurements, with the final assessment revealing a 33.2% decrease in S, a 27.7% decrease in G2, and rises of 20.8% in G1 and 63.3% in G0. Another extreme case revealed a significant shift between the first and second measurements in S (dropping by 12.5%) and G0 (increasing by 10.5%), with subsequent measurements creeping toward the initial measurement and thus yielding fewer extreme values, unlike the previously discussed subject. Its change is significantly less pronounced, suggesting it is more likely caused by a biological rather than analytical cause.

Finally, in two subjects, significant differences were observed in the fourth week, manifesting as spikes in Fig. 3. In one case, there was a decrease in S and G2 by 36%, along with increases in B (11%), G1 (14.6%) and a substantial 80.1% increase in G0. The other subject exhibited increases in S and G2 by 60.2% and 61.7%, respectively, coupled with decreases in G1 (12%) and a substantial 55.3% decrease in G0.

### Assessment of long-term stability

#### Five-year long-term stability

To examine the long-term stability, we recruited a single participant whose samples were collected every 3 months over a period of 5 years.

**Fig. 4:**
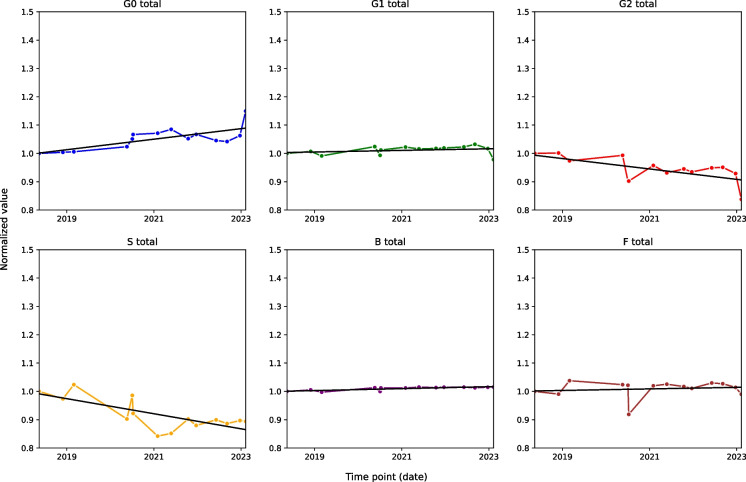
The plots illustrate the time-dependent trend for each derived glycan trait (G0, G1, G2, *S*, *B*, *F*) observed in a man over the 5-year period. The *x*-axis represents specific dates over time, while the *y*-axis displays the normalised values of relative abundance for each glycan trait. Data normalisation was conducted by dividing each data point value by its corresponding value at the initial point

The relative abundance of G0 increased by approximately 15% over the 5-year period, with a time effect of 0.000051. Conversely, the S trait decreased by around 10.5% during the entire period, with a time effect of − 0.000073. G2 showed the same pattern as the derived trait S with a time effect of − 0.000051. In contrast, traits G1 and F remained relatively stable throughout the observation period with time effects of 0.000007, showing minimal variation compared to other traits (Fig. 4). In addition, B displayed a smaller time effect of 0.000009 (Additional file 1: Table S5).

### Ten-year long-term stability

**Fig. 5:**
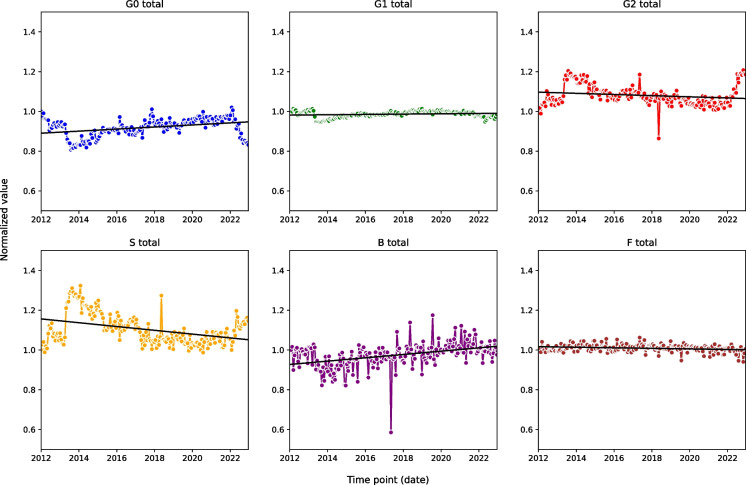
The plots illustrate the time-dependent trend for each derived glycan trait (G0, G1, G2, *S*, *B*, *F*) observed in a man over the 10-year period. The *x*-axis represents specific dates over time, while the *y*-axis displays the normalised values of relative abundance for each glycan trait. Data normalisation was conducted by dividing each data point value by its corresponding value at the initial point

In addition to the 5-year investigation, we recruited a single participant whose samples were collected every 3 weeks over a period of 10 years. Figure 5 presents noticeable trends in the derived glycan traits investigated over a 10-year period.

During the study period, G0 and B traits (time effects 0.000014 and 0.000023, respectively) exhibited an increase, suggesting an increase in glycan structures associated with these traits. Conversely, G2 and S (time effects of − 0.000008 and − 0.000026) decreased, indicating a reduction in digalactosylation and sialylation, respectively. Notably, G1 and F (time effects of 0.000002 and − 0.000004, respectively) remained remarkably constant throughout the 10-year period, showing much less pronounced time effects than other traits (Additional file 1: Table S5).

The most striking aspect of this dataset is the clear indication of two extended periods of changes (in late 2013 and 2022) that occurred in the opposite direction of ageing. These deviations were followed by a familiar upward trend, leading to the belief that their cause was biological rather than analytical.

## Discussion

In this paper, we presented an analysis of intermediate precision of the CGE-LIF method, analysed two different cohorts, evaluating short-term inter-individual variability, and analysed two different individuals, evaluating long-term intra-individual variability.

First, we explored the intermediate precision of the glycan clock of ageing. Understanding intermediate precision is crucial because it quantifies the impact of within-laboratory variations, including differences across various days, analysts, equipment used and other factors. In our experiment, we analysed three different pool samples throughout 26 “plates,” which represent individual days, with a single analyst and consistent equipment. During this period, we replaced the buffer and polymers according to the manufacturer’s instructions and prepared multiple batches of solutions, such as PBS. The directly measured glycan peaks, assessed using CV, demonstrated commendable precision across various samples. Our analysis revealed that the majority of glycan peaks (23 out of 27) exhibited variations below 10%. To the best of our knowledge, there were no previously established acceptance criteria for intermediate variation in the CGE-LIF method for glycan analysis. However, Rapčan et al. outlined acceptance criteria for the precision of the UPLC method in glycan analysis. According to these criteria, when the relative abundance (RA) of a peak is over 3%, the CV is expected to be under 5%. When the RA is between 3% and 0.5%, the CV is expected to be under 10%, and when the RA is under 0.5%, the CV is expected to be under 15% [33]. Bearing these criteria in mind, three peaks exhibited notable discrepancies. GP20 displayed the most significant deviation from the defined criteria. GP14 and GP1 also showed minor discrepancies, but they were marginal and very close to meeting the criteria, as their RAs hovered around 0.5% and their CVs were slightly above the 10% cutoff threshold. GP20 is a monogalactosylated bisected glycan. Changes in monogalactosylated glycans were not associated with an increase in biological age, while bisected glycans were, albeit their increase was far less pronounced than that of G0 increase and G2 reduction. It should be mentioned that GP20’s influence on the variation of B glycans is not significant, as it made up approximately 3% of the total B glycans. This becomes clear when evaluating the combined CV of bisected glycans with CVs below 2% across all three standards. As an agalactosylated and bisected glycan, GP14 is inherently linked to ageing; however, its impact is comparatively minor when compared to GP15, which significantly outweighs GP14 in abundance within agalactosylated and bisected glycans, contributing 35 to 40 times more in the samples analysed. Sialylated glycans, including GP1, are known for their slightly higher CV levels compared to other glycan peaks of comparable RA. Overall, all three peaks represent small RA peaks, each contributing less than 0.4% of the total IgG glycome. Even a slight change in these peaks (such as 0.1%) can lead to CVs as high as 25%. However, it is crucial to note that these criteria were designed for evaluating a single UPLC run, not for conducting a comprehensive 26-day assessment of intermediate precision of a CGE-LIF method. Given the significance of these findings, it is prudent to approach any comparison with the mentioned criteria cautiously. New criteria should be developed, particularly considering the high variation observed in GP20. Within plasma pool 6, GP6 showed much greater variation compared to the other two plasma pools. Peaks containing sialic acid (GP1-GP13) displayed more pronounced variation compared to their RA counterparts, which aligns with previous reports about sialic acid analytical stability issues [27]. This trend was also evident when examining glycan traits; F, representing 95% of the total sample, predictably exhibited the least variation, while S showed the highest variability due to previously identified factors. Of utmost significance, G0, which is the most frequently reported indicator of ageing among glycan traits of IgG glycome [34], exhibited low intermediate variation ranging from 1.24 to 1.59% across all three standards. Previous research did not provide intermediate precision results, opting instead to concentrate solely on calculating CVs across multiple batches within the same day and across multiple days. Furthermore, these studies utilised smaller data sets and employed different methodologies and analytical techniques [13].

After conducting the precision assessment, we proceeded to analyse the initial short-term cohort glycosylation patterns in healthy individuals, with a specific emphasis on exploring sex-specific differences and the impact of physiological processes. This approach was chosen in consideration of the documented influence of the menstrual cycle on glycosylation patterns. Given that oestrogen is recognized as a potent regulator, unlike testosterone, of IgG glycosylation in both men and women, it is notable that male oestrogen levels are significantly lower and more stable compared to those of women. Hence, based on previous confirmation of the menstrual cycle’s influence on IgG glycosylation in females, we expected that it might similarly impact IgG glycosylation over the 3-month observation period for the subjects compared to effects expected in men [22]. This can be attributed to a multitude of factors. We specifically examined young men, with a median age of 20, characterized by robust health and healthy habits. However, upon analysing data from female participants, we initially observed a statistically significant trend in trait F. Nonetheless, after correction for multiple testing, this significance was not sustained.

Subsequently, we investigated a cohort consisting of 70 women who were monitored weekly over 12 weeks. Our analysis during this 12-week monitoring period revealed statistically significant trends in both the traits G2, G0, and F among the participants. Considering the median age of 22.5 years among female participants, all with reported regular menstrual cycles, it is important to assess the menstrual cycle’s influence on IgG glycosylation. Research indicates fluctuations in all glycan traits, notably G2, G0, and S, during the menstrual cycle [22]. Although these fluctuations were described as minimal, their impact on stability assessment should be acknowledged, given their alignment with known effects of oestrogen [8, 35] and menstrual cycle IgG glycosylation [22]. Another crucial thing to note was that these effects were minimal and unlikely to significantly affect discrimination, especially for the F trait. Pathological changes typically induce much more pronounced variations compared to our reported observations, particularly in disease contexts. Change in F trait is especially interesting, as its fluctuation is not connected to the menstrual cycle.

In the CWPC cohort, we additionally investigated the potential occurrence of analytical errors by closely examining individual samples with extreme values. Out of the 795 measurements analysed, we detected two instances that seemed probable analytical errors, occurring at the fourth-time point. These outliers displayed a remarkable increase in trait S alongside a decrease in traits B and G2, with no subsequent measurements corroborating this pattern, and lacked a logical biological justification. The fact that we identified them is reassuring, as they were conspicuous and straightforward to detect, particularly as their origin is likely pre-analytical. This could potentially be attributed to sample switching, as each sample exhibited a glycan trait trajectory that was opposite to what was expected, and this reversal occurred by a comparable relative amount in each case. However, incorporating sample replicates in the analysis process could significantly mitigate such issues. Apart from the two aforementioned subjects, another intriguing case emerged within this cohort. This individual displayed a significant rise in G0 and G1, coupled with a notable decrease in G2 and S. While a discernible trend was evident, the pronounced disparities between the initial and subsequent points. This occurred in one of our older subjects (aged 40). Upon reviewing the subjects records, we observed a lengthening of their menstrual cycle from 27 to 31 days during the experiment. We also noticed some differences in the progesterone levels where during the second menstruation period, the concentrations 10 days before and 3 days before menstruation were 50.4 and 51.0 nmol/L, respectively, and in the very next cycle, they were 23.3 nmol/L 6 days before the cycle. Deciphering the underlying cause of these fluctuations in both glycans and progesterone levels is challenging given the available data. Nevertheless, it serves as compelling evidence supporting the biological explanation for the observed variations in glycosylation.

Over 5 years, changes in individuals’ glycomes were observed, marked by visible decreases in traits S and G2, and an increase in G0, along with a slight rise in B. All these changes were previously reported as part of the ageing process [36]. Reduction in glycan complexity is often connected to ageing. It is also often associated with age-associated conditions like cardiometabolic diseases and neurological disorders. Cardiometabolic diseases such as obesity, pre-, and hypertension often show increases in G0 and decreases in G2 and S in the case of hypertension [37, 38]. Rise in B was especially associated with obesity and central adiposity, something that many people experience as they age. Neurological conditions such as Alzheimer’s, Parkinson’s, and dementia typically exhibit decreases in the S glycan trait, with dementia also linked to an increase in B trait. Notably, deviations from these ageing-related trends were observed, particularly between 2020 and 2021, indicating accelerated ageing, coinciding with the documented impact of COVID-19, which was shown to increase G0 and F while reducing B and S, infection on IgG glycome composition [9]. These findings underscore the responsiveness of glycan traits to an individual’s physiological state and external factors.

Results from the 10-year follow-up of another individual’s glycome further support the notion of age-associated changes in IgG glycome composition [39]. Just like the 5-year subject, this subject experienced the same trends in their glycome. These trends are slightly less pronounced compared to the 5-year experiment because of the two notable interventions. These interventions stand out because the individual experienced significant decreases in traits associated with higher biological age (G0 and B, with the latter being less pronounced in the second instance), coupled with sharp increases in traits linked to lower biological age (S and G2, particularly pronounced in the first intervention). While we are not aware of the nature of these interventions, their impact is visible. Following the interventions, subsequent post-intervention samples reverted to a more expected ageing pattern, aligning with samples preceding these interventions. This consistency provides further confidence in attributing the observed changes to the assumed interventions rather than an analytical error. However, it is important to acknowledge that factors such as lifestyle adjustments, illnesses and environmental exposures can influence the relative abundance of glycan structures. Without detailed information about the specific nutritional, lifestyle or pharmacological interventions, pinpointing precise causes for specific changes remains challenging.

It is crucial to highlight that we meticulously accounted for various known factors that could influence glycosylation, such as sex, smoking habits, alcohol consumption, hormonal medications, oral contraceptives, other medications, menstrual cycle and the presence of any recent illnesses. Additionally, we intentionally recruited very young participants, with median ages of 20 for AMC and 22.5 for CWPC, aiming to minimize the impact of potential medical conditions on glycosylation patterns. However, it is important to acknowledge that certain uncontrollable variables, like fluctuations in diet, stress levels, and other external factors, which could have influenced our findings, were not closely monitored due to their inherent complexity in control. While our study made concerted efforts to mitigate these influences, the dynamic nature of human physiology and exposure to environmental factors underscores the ongoing need for vigilance and further exploration into the complexities of glycosylation.

Thus, it is important to underscore other limitations of our study. The primary concern is the absence of long-term subjects, as only two individuals were monitored over different time periods. This limited sample size for long-term observation constrains the generalizability of our findings to broader age-related changes in glycan composition. Furthermore, while we observed changes in glycan traits over time, the biological interpretation of these alterations remains challenging. Factors beyond the scope of our study, such as immune responses, genetic predispositions or other physiological processes, could influence glycan dynamics in ways not fully accounted for. Moreover, our understanding of interventions impacting glycan composition is restricted due to the lack of detailed information on the nutritional, lifestyle or pharmacological interventions observed in the study. Furthermore, the longitudinal study design, while valuable for tracking changes over time, poses inherent limitations, including participant attrition, potential biases and difficulty in controlling external variables that may influence glycan profiles. Lastly, we acknowledge the lack of female participants in the long-term experiment. Women who experience menopause tend to show accelerated ageing, and thus, their ageing is more likely to exhibit a sigmoidal rather than a linear pattern presented in our results [40]. The data gathered in the long-term studies cannot be applied to women. Addressing these limitations would be crucial for future research to enhance the robustness and applicability of glycan-based biomarkers in monitoring individual health trajectories accurately.

The introduction of the concept of the “glycan clock of ageing” has provided a fresh perspective, offering a new lens through which we can gain deeper insights into age-related research. Notably, IgG *N*-glycans have demonstrated the potential to reliably quantify molecular changes during ageing. This study highlights the remarkable precision and short-term stability of IgG glycome composition, coupled with a notable capacity to adapt and respond to physiological changes and environmental influences, especially apparent during the 10-year follow-up. In conclusion, our study elucidates the time-dependent dynamics and analytical precision of the glycan clock of ageing, highlighting its potential as a robust biomarker for monitoring individual health trajectories. By unveiling the intricate interplay between physiological processes, environmental factors, and glycan composition, our findings underscore the promising role of glycomics research in advancing personalised medicine and understanding the complexities of ageing.

## Supplementary Information

Below is the link to the electronic supplementary material.

Supplementary file1 (DOCX 33.6 KB)

## Data Availability

All generated data is available on reasonable request.
