# Supplementary material for: Glycan clock of ageing—analytical precision and time-dependent inter- and i-individual variability
Source: GeroScience. 2024 Jun 14;46(6):5781–96. doi: 10.1007/s11357-024-01239-4 (PMC11494675; doi:10.1007/s11357-024-01239-4)
Supplement: Supplementary file 1 — Supplementary file1 (DOCX 33.6 KB) [file 11357_2024_1239_MOESM1_ESM.docx]

Supplementary Table 1. The table provides formulas for calculating derived glycan traits from capillary gel electrophoresis with laser-induced fluorescence (CGE-LIF) electropherograms and ultra-high-performance liquid chromatography (UPLC) chromatograms. Each glycan peak is denoted by a GP followed by a number indicating its order on the corresponding graph. The corresponding letters (G0, G1, G2, S, B, F) represent distinct glycosylation traits: G0 for agalactosylation, G1 for monogalactosylation, G2 for digalactosylation, S for sialylation, B for bisecting N-acetylglucosamine, and F for core fucosylation in protein structures.

| Method | Glycan trait | Trait formula |
| --- | --- | --- |
| CGE-LIF | G0 | GP14+GP15+GP18 |
|  | G1 | GP16+GP17+GP19+GP20+GP21+GP22+GP23+GP24 |
|  | G2 | GP25+GP26+GP27 |
|  | S | GP1+GP2+GP3+GP4+GP5+GP6+GP7+GP8+GP9+GP10+GP11+GP12+GP13 |
|  | B | GP2+GP4+GP11+GP13+GP14+GP18+GP19+GP20+GP23+GP24+GP25+GP27 |
|  | F | GP3+GP4+GP7+GP8+GP12+GP13+GP15+GP18+GP21+GP22+GP23+GP24+GP26+GP27 |
| UPLC | G0 | GP1+GP2+GP3+GP4+GP6 |
|  | G1 | GP7+GP8+GP9+GP10+GP11 |
|  | G2 | GP12+GP13+GP14+GP15 |
|  | S | GP16+GP17+GP18+GP19+GP20+GP21+GP22+GP23+GP24 |
|  | B | GP3+GP6+GP10+GP11+GP13+GP15+GP19+GP22+GP24 |
|  | F | GP1+GP4+GP6+GP8+GP9+GP10+GP11+GP14+GP15+GP16+GP18+GP19+GP20+GP23+GP24 |

Supplementary Table 2. Time effect along with their corresponding standard error and (adjusted) p values on glycan traits over 90 days in healthy male individuals. The corresponding letters (G0, G1, G2, S, B, F) represent distinct glycosylation traits: G0 for agalactosylation, G1 for monogalactosylation, G2 for digalactosylation, S for sialylation, B for bisecting N-acetylglucosamine, and F for core fucosylation in protein structures.

| Glycan trait | Time effect | Standard error | p-value | Adjusted p-value |
| --- | --- | --- | --- | --- |
| G0 | -0.00135 | 0.00165 | 0.42454 | 0.63681 |
| G1 | -0.00163 | 0.00170 | 0.34990 | 0.59983 |
| G2 | 0.00174 | 0.00169 | 0.31835 | 0.59983 |
| S | 0.00111 | 0.00233 | 0.63907 | 0.76689 |
| B | 0.00244 | 0.00168 | 0.16495 | 0.51477 |
| F | -0.00115 | 0.00385 | 0.76882 | 0.76882 |

Supplementary Table 3. Time effect along with their corresponding standard error and (adjusted) p values on glycan traits over 90 days in the healthy female individuals. The corresponding letters (G0, G1, G2, S, B, F) represent distinct glycosylation traits: G0 for agalactosylation, G1 for monogalactosylation, G2 for digalactosylation, S for sialylation, B for bisecting N-acetylglucosamine, and F for core fucosylation in protein structures.

| Glycan trait | Time effect | Standard error | p-value | Adjusted p-value |
| --- | --- | --- | --- | --- |
| G0 | -0.00036 | 0.00096 | 0.70989 | 0.76882 |
| G1 | -0.00079 | 0.00113 | 0.48688 | 0.64917 |
| G2 | -0.00203 | 0.00146 | 0.17159 | 0.51477 |
| S | 0.00249 | 0.00202 | 0.22254 | 0.53410 |
| B | -0.00127 | 0.00073 | 0.08861 | 0.51477 |
| F | -0.00495 | 0.00236 | 0.04028 | 0.48341 |

Supplementary Table 4. Time effect along with their corresponding standard error and (adjusted) p values on glycan traits over 12 weeks in the menstrual cycle cohort. The corresponding letters (G0, G1, G2, S, B, F) represent distinct glycosylation traits: G0 for agalactosylation, G1 for monogalactosylation, G2 for digalactosylation, S for sialylation, B for bisecting N-acetylglucosamine, and F for core fucosylation in protein structures.

| Glycan trait | Time effect | Standard error | p-value | Adjusted p-value |
| --- | --- | --- | --- | --- |
| G0 | -0.02152 | 0.01002 | 0.03178 | 0.06357 |
| G1 | 0.00623 | 0.00561 | 0.26684 | 0.40026 |
| G2 | 0.01675 | 0.00650 | 0.00999 | 0.02998 |
| S | -0.00151 | 0.00934 | 0.87149 | 0.87149 |
| B | -0.00100 | 0.00251 | 0.69162 | 0.82994 |
| F | 0.00794 | 0.00257 | 0.00202 | 0.01211 |

Supplementary Table 5. The table provides a comprehensive overview of derived glycan traits over a 5- and 10-year period, incorporating follow-up times, glycan traits, and the time effect described through the slope of the trendline. The data was generated using a Python script. The corresponding letters (G0, G1, G2, S, B, F) represent distinct glycosylation traits: G0 for agalactosylation, G1 for monogalactosylation, G2 for digalactosylation, S for sialylation, B for bisecting N-acetylglucosamine, and F for core fucosylation in protein structures.

| Follow-up time | Glycan trait | Time effect/slope | Standard Error |
| --- | --- | --- | --- |
| 5 years | G0 | 0.000051 | 0.103193 |
|  | G1 | 0.000007 | 0.867004 |
|  | G2 | -0.000050 | 1.881824 |
|  | S | -0.000073 | 0.870229 |
|  | B | 0.000009 | 2.277605 |
|  | F | 0.000007 | 0.835279 |
| 10 years | G0 | 0.000014 | 0.672367 |
|  | G1 | 0.000002 | 0.947782 |
|  | G2 | -0.000008 | 1.219469 |
|  | S | -0.000026 | 1.075918 |
|  | B | 0.000023 | 1.557251 |
|  | F | -0.000004 | 0.576486 |
